# The schizophrenia and gut microbiota: A bibliometric and visual analysis

**DOI:** 10.3389/fpsyt.2022.1022472

**Published:** 2022-11-15

**Authors:** Chao Yang, Xiaoxiao Lin, Xianteng Wang, Huanzhong Liu, Jinyu Huang, Shuai Wang

**Affiliations:** ^1^Department of Psychiatry, Beijing Luhe Hospital, Capital Medical University, Beijing, China; ^2^The Fourth School of Clinical Medicine, Zhejiang Chinese Medical University, Hangzhou, China; ^3^Guangdong Key Laboratory for Biomedical Measurements and Ultrasound Imaging, School of Biomedical Engineering, Shenzhen University Health Science Center, Shenzhen, China; ^4^Shenzhen Institute of Translational Medicine, Shenzhen Second People’s Hospital, The First Affiliated Hospital of Shenzhen University, International Cancer Center of Shenzhen University, Shenzhen, China; ^5^Department of Psychiatry, Chaohu Hospital, Anhui Medical University, Chaohu, China; ^6^Department of Cardiology, Affiliated Hangzhou First People’s Hospital, Zhejiang University School of Medicine, Hangzhou, China; ^7^Department of Translation Medicine Center, Affiliated Hangzhou First People’s Hospital, Zhejiang University School of Medicine, Hangzhou, China

**Keywords:** gut microbiota, bibliometric analysis, gut-brain axis, schizophrenia, antipsychotics

## Abstract

**Background:**

Many studies have explored the link between the gut microbiota and schizophrenia. To date, there have been no bibliometric analyses to summarize the association between the gut microbiota and schizophrenia. We aimed to conduct a bibliometric study of this association to determine the current status and areas for advancement in this field.

**Materials and methods:**

Publications related to the gut microbiota and schizophrenia were retrieved from the Web of Science Core Collection (WoSCC). The WoSCC literature analysis wire and VOSviewer 1.6.16 were used to conduct the analysis.

**Results:**

In total, 162 publications were included in our study. The publications generally showed an upward trend from 2014. A total of 873 authors from 355 organizations and 40 countries/regions contributed to this field. The leading authors were Timothy Dinan, John F Cryan, and Emily Severance. The leading institutions were Johns Hopkins University, the University College Cork, and the University of Toronto. The most productive countries were the United States (US), China, and Canada. In total, 95 journals contributed to this field. Among them, the top three productive journals were Schizophrenia Research, Progress in Neuro Psychopharmacology Biological Psychiatry, and Frontiers in Psychiatry. The important keywords in the clusters were gut microbiome, bipolar disorder, schizophrenia, antipsychotics, weight gain, metabolic syndrome, gut-brain axis, autism, depression, inflammation, and brain.

**Conclusion:**

The main research hotspots involving the connection between schizophrenia and the gut microbiota were the characteristics of the microbiota composition in schizophrenia patients, the gut-brain axis, and microbial-based interventions for schizophrenia. The studies about the association between gut microbiota and schizophrenia are limited, and more studies are needed to provide new insights into the gut microbiota in the pathogenesis and treatment of schizophrenia.

## Introduction

Schizophrenia is a serious psychiatric illness that affects approximately one in a hundred people worldwide (1–5). The rate of early mortality is 2- to 3-fold higher in patients with schizophrenia than that in the general population (6–10). The current interventions for schizophrenia are mainly antipsychotic medications, including olanzapine, risperidone, aripiprazole, and clozapine; modified electroconvulsive therapy (MECT); and repetitive transcranial magnetic stimulation (rTMS). Drugs for schizophrenia may have some side effects, including weight gain and metabolic disturbances (11–14).

In recent years, the link between schizophrenia and the gut microbiota has received increasing attention. Recent studies have shown that the gut microbiota can modulate brain function through the gut-brain axis (15–19). The gut microbiota may provide a possible mechanism for the development of schizophrenia. The alterations and dysbiosis in the function and composition of the gut microbiome are found to be associated with schizophrenia though the modulation of glutamatergic neurotransmission metabolism and tryptophan–kynurenine metabolism (18, 20–23). The mice received the gut microbiome from patients with schizophrenia displayed altered lipid metabolism and amino acid, and decreased brain glutamate and disruptions in the glutamate–glutamine–GABA cycle, which were implicated in the pathophysiology of schizophrenia (20–22). In mice received the gut microbiome from patients with schizophrenia, the serotonin pathway of tryptophan catabolism was markedly reduced, while the Kyn–Kyna pathway of tryptophan catabolism was increased, which is related to schizophrenia by the modulation of tryptophan–kynurenine metabolism (23). Moreover, microbial-based therapies may be effective for antipsychotic-induced weight gain and metabolic disturbances. Emerging preclinical and clinical studies have demonstrated potential associations between schizophrenia and the gut microbiota. Bibliometric analysis has been widely used to determine the current status and explore developmental trends by the quantitative analysis of patterns in the scientific literature. By this method, researchers can understand the range of research topics and predict future directions in specific field. To date, there have been no bibliometric analyses to summarize the link between the gut microbiota and schizophrenia. We aimed to conduct a bibliometric study of the association between gut microbiota and schizophrenia to determine the current status and areas for advancement in this field.

## Materials and methods

In our study, publications about schizophrenia and the gut microbiota were downloaded from the WoSCC. The following search terms were used: TS = (microbiome* OR flora microbiot* OR bacteria OR microflora) AND TS = (intestin* OR gut OR gastro-intestin* OR gastrointestin*) AND TS = (schizophrenia OR “schizoaffective disorder” OR “schizophreniform disorder” OR “first episode psychosis” OR “schizophrenia spectrum disorder”). The search results were confined by the publication date from the inception of the study to 30 May 2022; types of publications, including articles and reviews; and English language. In our study, we conducted a literature search and screening according to the Preferred Reporting Items for Systematic Reviews and Meta-Analyses (PRISMA) guidelines (24).

### Data collection and analysis

The *h*-index was used to evaluate the citations of the researcher’s publications. The WoSCC literature analysis wire and VOSviewer 1.6.16 were used to conduct the analysis. The WoSCC literature analysis wire was used to analyze the publication years, categories, document types, the distribution of authors, institutions, countries/regions, and *h*-index. VOSviewer1.6.16 software was used to conduct assessments of the coauthorship of authors, keywords, countries/regions, and institutions. The total link strength (TLS) was used to evaluate the cooperation relationship. In the keyword co-occurrence analysis, we merged the synonyms of “induced weight-gain,” weight-gain and “weight gain” to the term “weight gain”; “probiotic supplementation” and “probiotics” into “probiotics”; “autism spectrum disorders,” “autism spectrum disorder,” and “autism” into the term “autism”; and “microbiome,” “microbiota,” “intestinal microbiota,” “gut microbiome,” “gut microbiota,” and “fecal microbiota” into the term “gut microbiota.”

## Results

### Publication output

**FIGURE 1:**
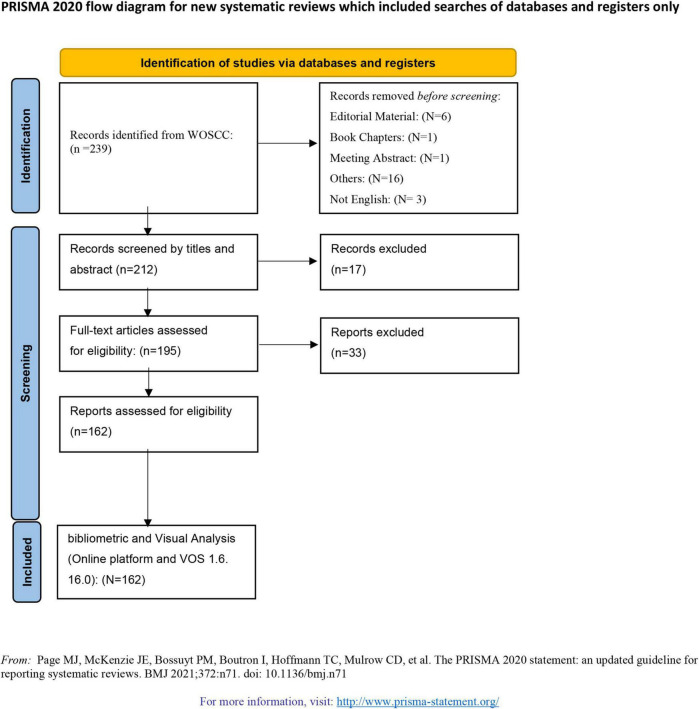
PRISMA flowchart.

**FIGURE 2:**
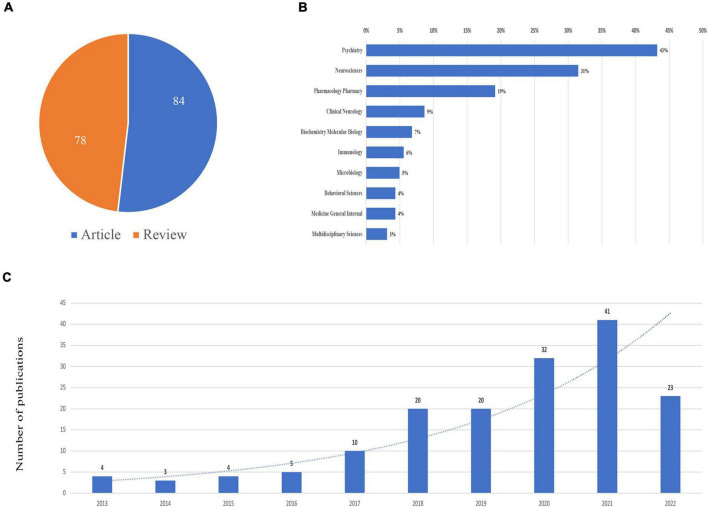
Publications on schizophrenia and gut microbiota from inception to 2022. **(A)** Literature types distribution. **(B)** Subject categories distribution. **(C)** Annual publications quantitative distribution.

A total of 162 publications containing 78 reviews and 84 articles were included, as shown in Figure 1. The publications generally showed an upward trend from 2014. The top subject categories were psychiatry with 70 publications and neurosciences with 51 publications (Figure 2).

### Distribution of authors

**FIGURE 3:**
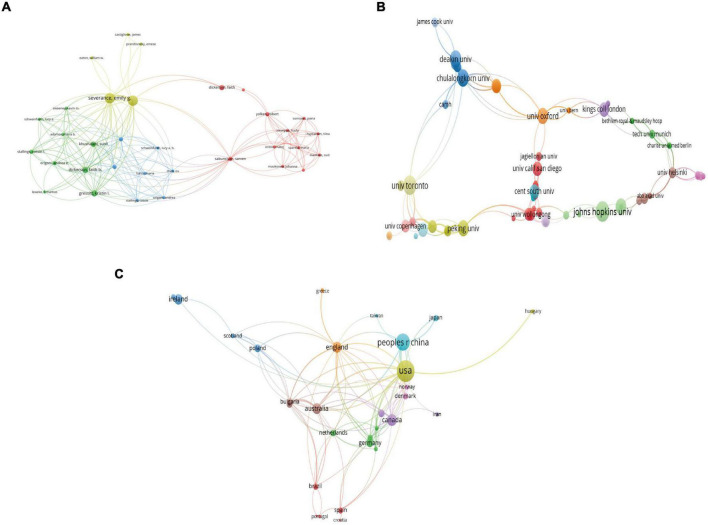
Visualization knowledge maps of the co-authorship. **(A)** Authors. **(B)** Organizations. **(C)** Countries/regions.

In total, 873 authors contributed to this field. Timothy Dinan was the leading author who had 13 publications with 1,933 citations and an *h*-index of 12, followed by John F Cryan who had 12 publications with 1,929 citations and an *h*-index of 12, Emily Severance who had 11 publications with 580 citations and an *h*-index of 9, Robert Yolken who had 11 publications with 580 citations and an *h*-index of 9, and Michael Maes who had 7 publications with 160 citations and an *h*-index of 7. Figure 3A shows the coauthorship map of the authors. The Severance Emily and Yolken Robert had the highest TLS, indicating that they participated in the most collaborations with other authors.

### Distribution by countries/regions and institutions

All publications were from 355 organizations and 40 countries/regions. The top three most productive institutions were Johns Hopkins University with 14 publications and 931 citations, University College Cork with 14 publications and 1,942 citations, and the University of Toronto with 10 publications and 341 citations. Figure 3B shows the coauthorship map of institutions. Regarding countries/regions, the United States (US) had the most publications with 59 documents, followed by China with 37 documents, Canada with 18 documents, Australia with 15 documents, and England with 15 documents. Figure 3C shows the coauthorship map of the countries/regions. Table 1 shows the top 10 high-yield institutions, authors, and countries/regions.

**TABLE 1:** Ranking of the top 10 authors, institutions, and countries.

Items	Publications					Co-authorship maps		
	Rank	Country	Number	Citations	H-index	Rank	Name	Total link strength
Country	1	USA	59	2521	27	1	USA	72
	2	Peoples R China	37	978	15	2	Australia	37
	3	Canada	18	565	10	3	Canada	35
	4	Australia	15	335	10	4	England	34
	5	England	15	328	9	5	Peoples R China	34
	6	Ireland	14	1942	12	6	Bulgaria	25
	7	Germany	10	494	7	7	Thailand	25
	8	Italy	8	106	5	8	Germany	19
	9	Poland	8	162	7	9	Switzerland	16
	10	Bulgaria	7	160	7	10	Norway	14
Institution	1	Johns Hopkins University	14	931	10	1	Deakin University	42
	2	University College Cork	14	1942	12	2	University of Toronto	38
	3	University of Toronto	10	341	8	3	Chulalongkorn University	36
	4	University of California system	9	390	8	4	Medical University Plovdiv	36
	5	Central South University	7	162	5	5	University of Helsinki	30
	6	Chulalongkorn University	7	160	7	6	Xi An Jiao Tong University	30
	7	Deakin University	7	150	7	7	University of Chinese Academy of Sciences	29
	8	Medical University Plovdiv	7	160	7	8	University of Copenhagen	23
	9	Centre For Addiction Mental Health Canada	6	103	5	9	BGI Shenzhen	21
	10	Harvard University	6	265	6	10	China National Genebank	21
Author	1	Dinan, Timothy	13	1933	12	1	Severance, Emily	54
	2	Cryan, John F	12	1929	12	2	Yolken, Robert	54
	3	Severance, Emily	11	580	9	3	Dinan, Timothy	43
	4	Yolken, Robert	11	580	9	4	Cryan, John F	38
	5	Maes, Michael	7	160	7	5	Gressitt, Kristin I	35
	6	Clarke, Gerard	6	1690	6	6	Knight, Rob	34
	7	Knight, Rob	5	270	5	7	Kosciolek, Tomasz	34
	8	Kosciolek, Tomasz	5	270	5	8	Dickerson, Faith B	32
	9	Misiak, BłaŻej	5	91	4	9	Khushalani, Sunil	30
	10	Samochowiec, Jerzy	5	91	4	10	Chen, Jun	28

### Distribution by journal

**FIGURE 4:**
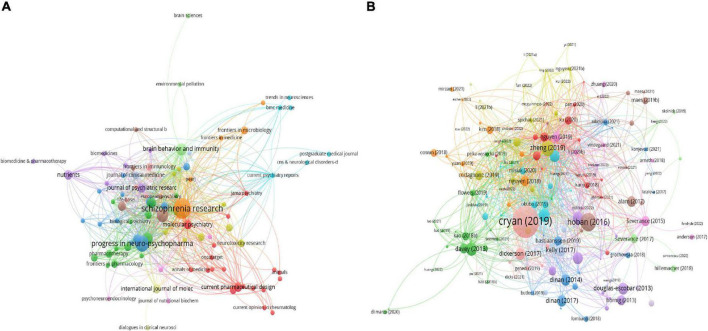
Visualization knowledge maps of citations. **(A)** Journals. **(B)** Documents.

In total, 95 journals contributed to this field. Among them, the top three productive journals were Schizophrenia Research with 14 documents and 809 citations, Progress in Neuro Psychopharmacology Biological Psychiatry with 9 documents and 115 citations, and Frontiers in Psychiatry with 6 documents and 51 citations. Figure 4A and Table 2 show the coauthorship map of journals and the top 10 high-yield journals.

**TABLE 2:** Ranking of the top 10 journals based on publications.

Ranking	Journal name	Country	Counts	Citation	H-index
1	Schizophrenia Research	Netherlands	14	809	10
2	Progress in Neuro Psychopharmacology Biological Psychiatry	England	9	115	7
3	Frontiers in Psychiatry	Switzerland	6	51	4
4	Brain Behavior and Immunity	United States	5	290	4
5	Neuroscience and Biobehavioral Reviews	England	4	81	3
6	Translational Psychiatry	England	4	413	3
7	Current Opinion in Psychiatry	United States	3	28	2
8	Current Pharmaceutical Design	U Arab Emirates	3	40	2
9	International Journal of Molecular Sciences	United States	3	12	1
10	Journal of Psychiatric Research	England	3	131	3

### Analysis of highly cited documents

Table 3 shows the top twenty most-cited publications (23, 25–43). The first highest-cited article with 918 citations was published in Physiological Reviews and authored by Cryan et al. in 2019. This review demonstrates that the gut microbiota is associated with many diseases, including schizophrenia and Parkinson’s disease. Animal models have been paramount in linking the activation of microglia induced by the microbiome to the regulation of fundamental neural processes, and translational human studies are ongoing and will greatly enhance this field. The second most-cited article, with 307 citations, was published in Translational Psychiatry and authored by Hoban et al. in 2016. They found that the microbiome was necessary for dynamic and appropriate regulation of myelin-related genes and may be a therapeutic target for schizophrenia and autism. The third most-cited article, with 223 citations, was published in Science advances and authored by Zheng et al in 2019. They found that patients with schizophrenia exhibited marked disturbances in gut microbial composition and a decreased microbiome α-diversity index. The abundances of organisms assessed in microbial panel, including *Aerococcaceae*, *Rikenellaceae*, *Pasteurellaceae*, *Brucellaceae*, and *Bifidobacteriaceae*, decreased in patients with schizophrenia. Their results demonstrated that the microbiome may alter neurologic function and neurochemistry, which is related to the pathology of schizophrenia. The fourth most-cited article, with 155 citations, was published in Schizophrenia research and authored by Schwarz et al. in 2018. They found that *Lactobacillus* abundance was elevated in patients with first-episode psychosis (FEP) and was related to the severity of schizophrenia. Their results showed that microbiota alterations benefited remission and treatment response in schizophrenia patients. Figure 4B show the coauthorship map of documents.

**TABLE 3:** Ranking of the top 20 highest cited references.

Rank	Title	Journal	Total citations	Publication year	First author
1	The Microbiota-Gut-Brain Axis	Physiological Reviews	918	2019	John F. Cryan
2	Regulation of prefrontal cortex myelination by the microbiota	Translational Psychiatry	307	2016	A E Hoban
3	The gut microbiome from patients with schizophrenia modulates the glutamate-glutamine-GABA cycle and schizophrenia-relevant behaviors in mice	Science Advances	223	2019	Peng Zheng
4	Analysis of microbiota in first episode psychosis identifies preliminary associations with symptom severity and treatment response	Schizophrenia Research	155	2018	Emanuel Schwarz
5	Discordant patterns of bacterial translocation markers and implications for innate immune imbalances in schizophrenia	Schizophrenia Research	147	2013	Emily G Severance
6	The microbiome, immunity, and schizophrenia and bipolar disorder	Brain Behavior and Immunity	132	2017	Faith Dickerson
7	Cross Talk: The Microbiota and Neurodevelopmental Disorders	Frontiers In Neuroscience	129	2017	John R Kelly
8	Analysis of gut microbiota diversity and auxiliary diagnosis as a biomarker in patients with schizophrenia: A cross-sectional study	Schizophrenia Research	127	2018	Yang Shen
9	Autoimmune diseases, gastrointestinal disorders and the microbiome in schizophrenia: more than a gut feeling	Schizophrenia Research	124	2016	Emily G Severance
10	Brain-Gut-Microbiota Axis and Mental Health	Psychosomatic Medicine	122	2017	Timothy G Dinan
11	Genomics of schizophrenia: time to consider the gut microbiome?	Molecular Psychiatry	113	2014	Timothy G Dinan
12	Microbiome, inflammation, epigenetic alterations, and mental diseases	American Journal of Medical Genetics Part B-Neuropsychiatric Genetics	106	2017	Reza Alam
13	The Antipsychotic Olanzapine Interacts with the Gut Microbiome to Cause Weight Gain in Mouse	Plos One	98	2014	Andrew P Morgan
14	Overview and systematic review of studies of microbiome in schizophrenia and bipolar disorder	Journal Of Psychiatric Research	97	2018	Tanya T Nguyen
15	Gastroenterology Issues in Schizophrenia: Why the Gut Matters	Current Psychiatry Reports	96	2015	Emily G Severance
16	The role of microbes and autoimmunity in the pathogenesis of neuropsychiatric illness	Current Opinion in Rheumatology	92	2013	Mady Hornig
17	Programming Bugs: Microbiota and the Developmental Origins of Brain Health and Disease	Biological Psychiatry	88	2019	Martin G Codagnone
18	Differences in gut microbiome composition between persons with chronic schizophrenia and healthy comparison subjects	Schizophrenia Research	85	2019	Tanya T Nguyen
19	Metagenome-wide association of gut microbiome features for schizophrenia	Nature Communications	84	2020	Feng Zhu
20	Transplantation of microbiota from drug-free patients with schizophrenia causes schizophrenia-like abnormal behaviors and dysregulated kynurenine metabolism in mice	Molecular Psychiatry	80	2020	Feng Zhu

### Analysis of keywords co-occurrence clusters

**FIGURE 5:**
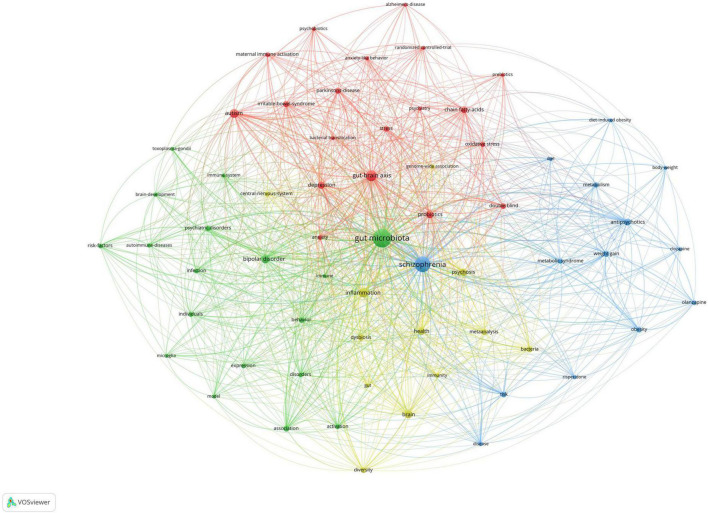
Visualization of keyword co-occurrence analysis.

Figure 5 shows the co-occurrence map of keywords, which indicates four research directions. The cluster represented in green includes the important keywords of gut microbiome and bipolar disorder. The cluster represented in blue includes the important keywords of schizophrenia, antipsychotics, weight gain, and metabolic syndrome. The cluster represented in red includes the important keywords of gut-brain axis, autism, and depression. The cluster represented in yellow includes the important keywords of inflammation and brain.

## Discussion

### General information

To the best of our knowledge, this is the first bibliometric study to explore the link between schizophrenia and the gut microbiota. In total, 162 publications were included in our study. The publications generally showed an upward trend from 2014. The most top subject categories were psychiatry with 70 publications. A total of 873 authors from 355 organizations and 40 countries/regions contributed to this field. The leading authors were Timothy Dinan who had 13 publications with 1,933 citations and an *h*-index of 12, John F Cryan who had 12 publications with 1,929 citations and an *h*-index of 12, and Emily Severance who had 11 publications with 580 citations and an *h*-index of 9. The Severance Emily and Yolken Robert participated in the most collaborations with other authors. The leading institutions were Johns Hopkins University with 14 publications, the University College Cork with 14 publications, and the University of Toronto with 10 publications. The most productive countries were the US with 59 documents, China with 37 documents, and Canada with 18 documents. In total, 95 journals contributed to this field. Among them, the top three productive journals were Schizophrenia Research with 14 documents, Progress in Neuro Psychopharmacology Biological Psychiatry with 9 documents, and Frontiers in Psychiatry with 6 documents. There are four research directions in the clusters. The cluster represented in green includes the important keywords of gut microbiome and bipolar disorder. The cluster represented in blue includes the important keywords of schizophrenia, antipsychotics, weight gain, and metabolic syndrome. The cluster represented in red includes the important keywords of gut-brain axis, autism, and depression. The cluster represented in yellow includes the important keywords of inflammation and brain.

In the field of schizophrenia and gut microbiota, most of the articles were published in the US, and the percentage of articles from the US was 38% (155/388). Among the top ten institutions, six were located in the US. Among the top ten productive authors, five were from the US. The prominent country in this field was the US. The number of studies from other countries/regions should be improved.

### Areas of advancement and hotspots

Based on the important keywords and the top 20 most-cited references, the research hotspots and areas for advancement in research on the link between schizophrenia and the gut microbiota were found to be as follows: (1) The characteristics of the microbiota composition in schizophrenia. In the top 20 most-cited references, 5 publications explored the characteristics of the microbiota composition in schizophrenia patients. A systematic review (44) demonstrated that there were significant differences in beta diversity but not alpha diversity between patients with and without schizophrenia. Zheng et al. showed that *Veillonella* abundance was significantly higher while *Ruminococcus* and *Roseburia* abundances were significantly lower in patients with schizophrenia. Germ-free mice that received fecal microbiota transplantation (FMT) from the microbiota of schizophrenia patients had higher glutamine and GABA levels and lower glutamate levels in the hippocampus. There was considerable discord between these results due to factors including small sample sizes, potential confounders, and the measurement methods. More large-scale prospective studies should be conducted to identify whether specific microbiome compositions are associated with an increased risk of developing schizophrenia. (2) The role of the gut-brain axis. In the top 20 most-cited references, 7 publications explored the gut-brain axis and its role in the association between schizophrenia and the gut microbiota, and the important keywords gut-brain axis, inflammation, and brain were in the cluster represented in blue. The gut microbiota was found to be associated with schizophrenia *via* processes involved in the gut-brain axis, including immune-regulating pathways, neurotransmitter synthesis, the production of bioactive microbial metabolites, and tryptophan metabolism (34, 45–48). Among the involved molecular features, immune mediators are the most important intermediaries between schizophrenia and the gut microbiota (49–53). Gastrointestinal symptoms were found to be chronic comorbidities observed in schizophrenia patients. The gut microbiota may play an important role in the development of the neuroimmune system, neuronal remodeling, synaptic pruning, and myelination. The pathophysiology of schizophrenia is associated with immune system alterations. The gut microbiota is also involved in the activation and maturation of microglia, which may play an important role in the development of schizophrenia (54–56). (3) Microbial-based interventions for schizophrenia. In the top 20 most-cited references, 7 publications explored the potential role of targeting the gut microbiota as an intervention for schizophrenia, and the important keywords antipsychotics, weight gain and metabolic syndrome were in the cluster represented in blue. Prebiotics and probiotics are potential treatments to improve cognition, neural activity, anxiety, and gastrointestinal symptoms for patients with schizophrenia (41, 57–60). In addition, prebiotics and probiotics could alleviate the metabolic side effects induced by antipsychotics (61–67). More studies should be performed to explore microbiome-mediated treatment for ameliorating cognitive dysfunction and antipsychotic-associated weight gain.

There are some limitations to our study. First, all publications were from the WoSCC database because it was the best citation-based database and was the most widely used in bibliometric and visual analysis. Second, only publications in English were included in our study. Additionally, the literature produced in 2022 was not fully assessed because of the study cut-off time.

### Future directions

The studies about the association between gut microbiota and schizophrenia are limited although the gut microbiota may play an important role in the pathogenesis and treatment of schizophrenia. More studies are needed in the future. Most of existing studies are of cross-sectional design, so it is challenging to establish the causality. Further researches should focus on the role of gut microbiota in the pathogenesis of schizophrenia and the effective treatment for schizophrenia using data analysis approaches that deal more effectively to avoid confounding factors.

## Conclusion

The main research hotspots regarding the connection between schizophrenia and the gut microbiota were the characteristics of the microbiota composition in schizophrenia patients, the gut-brain axis, and microbial-based interventions for schizophrenia. The studies about the association between gut microbiota and schizophrenia are limited, and more studies are needed to provide new insights into the gut microbiota in the pathogenesis and treatment of schizophrenia.

## Data availability statement

The original contributions presented in this study are included in the article/supplementary material, further inquiries can be directed to the corresponding authors.

## Author contributions

CY, XW, HL, and XL were responsible for the data collection, investigation, figures and tables construction and writing the original draft. SW and JH contributed to the discussion and final review and editing. All authors reviewed and edited the final manuscript.
